# Fractalkine is expressed in the human ovary and increases progesterone biosynthesis in human luteinised granulosa cells

**DOI:** 10.1186/1477-7827-9-95

**Published:** 2011-06-30

**Authors:** Shuo Huang, Ping Zhao, Liying Yang, Yuan Chen, Jie Yan, Enkui Duan, Jie Qiao

**Affiliations:** 1 Reproductive Medical Center, Peking University Third Hospital, Beijing, People's Republic of China; 2 State Key Laboratory of Reproductive Biology, Institute of Zoology, Chinese Academy of Sciences, Beijing, People's Republic of China

## Abstract

**Background:**

Recent evidence from rodent ovaries has demonstrated expression of fractalkine and the existence of fractalkine receptor, and showed that there is a significant increase in steroidogenesis in response to fractalkine, yet the role of fractalkine and CX3CR1 in the human ovary is still unknown. This study aimed to determine the expression levels of fractalkine and CX3CR1 in the human ovary and to investigate their roles in sexual hormone biosynthesis by human luteinising granulosa cells. This is the first detailed report of fractalkine and CX3CR1 expression and function in the human ovary.

**Methods:**

Fractalkine and CX3CR1 expression levels were measured by immunohistochemistry using ovarian tissue from pathological specimens from five individuals. Granulosa cells were obtained from patients during IVF treatment. They were cultured and treated with increasing doses of hCG with or without fractalkine. Media were collected to detect estradiol and progesterone by chemiluminescence. StAR, 3-βHSD and CYP11A expression were determined in granulosa cells treated with or without fractalkine by real-time RT-PCR.

**Results:**

Fractalkine and CX3CR1 were expressed in the human ovary and in luteinising granulosa cells. However, fractalkine expression was stronger in luteinising granulosa cells. Treatment with fractalkine augmented hCG stimulation of progesterone production in a dose-dependent manner with concomitant increases in transcript levels for key steroidogenic enzymes (StAR, 3-βHSD and CYP11A) but had no effect on estradiol biosynthesis(*P *< 0.05).

**Conclusions:**

Fractalkine and CX3CR1 were found to express in human ovary and luteinising granulosa cells. Fractalkine can increase the biosynthesis of progesterone in a dose-dependent manner by enhancing transcript levels of key steroidogenic enzymes.

## Background

Previous studies have demonstrated the importance of multiple intraovarian ligand-receptor signalling systems in modulating ovarian function. These factors play paracrine and/or autocrine role(s) in oocyte maturation, ovulation, and luteinisation [1,2]. A critical feature of ovarian function is the differentiation of the ovulatory follicle into the corpus luteum, which produces steroid hormones (estradiol, progesterone) that are required for the initiation and maintenance of pregnancy [3]. Fractalkine was found to be localised in rat ovaries and excreted as an autocrine/paracrine factor to increase hCG (human chorionic gonadotropin) stimulation of progesterone [4].

Fractalkine is a chemokine that is synthesised at the sites of inflammation and is known to be a major regulatory protein for leukocyte recruitment and trafficking. More than 40 chemokines have been identified to date, and they are subdivided into four subfamilies, C-, CC-, CXC-, and CX3C-chemokines [5]. Fractalkine (also known as CX3CL1 or neurotactin) is the only member of a subclass of chemokines designated as the CX3C family. Recombinant fractalkine was found to activate a seven transmembrane receptor CX3CR1 [6].

DNA microarray analysis of mice treated with gonadotropin indicated that there were increases in the expression of ovarian fractalkine transcripts after a single injection of hCG, which induced ovulation. Further, transcript levels for the receptor CX3CR1 displayed minimal changes during gonadotropin treatment. Further research in rats has shown that fractalkine is localised in cumulus, mural granulosa, and theca cells, as well as in the oocytes, whereas CX3CR1 was found in the same cells, except for the oocytes. hCG could induce fractalkine transcripts in different ovarian compartments, with the highest increases found in granulosa cells. In the cultured granulosa cells of rats, treatment with fractalkine increased hCG stimulation of progesterone [4]. It has yet to determine the expression and roles of fractalkine in human.

To better understand what paracrine and/or autocrine role(s) fractalkine plays in human ovarian function, we designed the current study to examine the expression of fractalkine and its receptor CX3CR1 in human ovaries and to explore the effects of fractalkine on progesterone and estradiol production in human luteinised granulosa cells *in vitro*.

## Methods

### Subjects

The experimental protocol was approved by the ethics committee of Peking University Health Science Center. Paraffin sections of human ovary tissues were obtained from the Pathology Department of Peking University Third Hospital from five patients aged 32-49 years. All five patients had suffered ovary excision because of benign ovarian lesions (Table 1). Human luteinised granulosa cells (GCs) were isolated from follicular aspirates of women undergoing *in vitro *fertilisation and embryo transfer (IVF-ET) at the Reproductive Center of Peking University Third Hospital. All twenty seven women aged 25-39 years who had regular menstrual cycles underwent IVF-ET only because of male factors. Women with a diagnosis of polycystic ovarian syndrome or endometriosis were excluded.

**Table 1:** Information about the patients collected for immunohistochemical study

	Age	diagnosis
Patient 1	39	dermoid cyst
Patient 2	42	endometrial cyst
Patient 3	32	dermoid cyst
Patient 4	37	serous cystadenoma
Patient 5	49	endometrial cyst

All five patients underwent unilateral ovary excision

### Immunohistochemistry

To identify and locate the expression of fractalkine and CX3CR1, after deparaffinisation and rehydration, 5-μm sections of ovaries were pretreated in EDTA buffer (pH 8.0) for 15 min at 95°C to retrieve antigens. Tissue sections were then immersed in 1.5% peroxide/methanol for 15 min to remove endogenous peroxidase activity followed by blocking in 5% BSA for 60 min. Slides were incubated with polyclonal rabbit anti-mouse/human/rat fractalkine (eBioscience Inc, San Diego, United States) at a 1:200 dilution or polyclonal rabbit anti-mouse/human/rat CX3CR1 (eBioscience Inc, San Diego, United States) at a 1:200 dilution for 18 h at 4°C. After three washes in phosphate-buffered saline (PBS), slides were incubated with biotin-conjugated anti-rabbit secondary antibodies (Santa Cruz Biotechnology Inc., Santa Cruz, United States) for 1 h at 37°C. Tissue sections were then washed three times and incubated with horseradish peroxidase-conjugated streptavidin (Santa Cruz Biotechnology Inc., Santa Cruz, United States) for 30 min at 37°C. Signals were developed using the diaminobenzidine kit (Santa Cruz Biotechnology Inc., Santa Cruz, United States), and cell nuclei were stained using hematoxylin solution (Sigma-Aldrich Inc., St. Louis, United States). For negative controls, the primary antibodies were replaced by PBS.

### Isolation and culture of granulosa cells

Follicular granulosa cells (GCs) were aspirated from each patient undergoing IVF-ET, washed in PBS, and centrifuged over 45% Percoll (Sigma-Aldrich Inc., St. Louis, United States) to remove red blood cells. The number of granulosa cells that could be aspirated from one woman is about from 2 × 10^6 ^to 3 × 10^6^. GCs were washed again with McCoy's 5a media (Modified; Invitrogen) and then cellular deposits were collected.

GCs were resuspended in McCoy's 5a medium supplemented with 10^-7 ^M androstenedione, 100 U/ml penicillin, and 100 μg/ml streptomycin and cultured in plates treated under different conditions. Cells were grown on the glass coverslips for 24 h in McCoy's 5a media with 5% fetal bovine serum for immunofluorescence. Cells were cultured in 48-well plates (Nunclon, Roskilde, Denmark) in serum-free McCoy's 5a media treated with increasing doses of hCG (Merck Serono, Geneva, Swiss) with or without recombinant human fractalkine (R&D Systems, Emeryville, United States) at 100 ng/ml to determine the possible effects of fractalkine on progesterone and estradiol production. To test the dose dependent effects of fractalkine, cells were treated with 20 ng/ml hCG and varying concentrations of recombinant fractalkine. Cells were cultured in 6-well plates treated with 20 ng/ml hCG with or without recombinant human fractalkine (100 ng/ml) for 48 hours for further real-time RT-PCR analysis. The number of the cells seeded in the 48-well plates and in the 6-well plates is separately 1.5 × 10^5 ^per well and 1 × 10^6 ^per well.

### Immunofluorescence

Cells were grown and attached well on glass coverslips, as confirmed by microscopic observation. They were then fixed in methanol for 30 min at 4°C and washed in PBS. The cells were subsequently subjected to triton-X 100 for 30 min and then to 5% BSA blocking for 60 min. The cells were incubated with polyclonal rabbit anti-mouse/human/rat fractalkine and polyclonal rabbit anti-mouse/human/rat CX3CR1 diluted to 1:200 with blocking solution overnight at 4°C. This was followed by three washes with PBS containing 0.5% Tween (PBST). Then the cells were incubated with secondary goat anti-rabbit fluorescence isothiocyanate (FITC)-conjugated antibodies diluted to 1:100 for 1 hour at 37°C. After rinsing, cell nuclei were stained with PI for 5 min. Finally, the slides were sealed with 50% glycerol and observed using a confocal laser scanning microscope (Leica, Germany).

### Measurement of steroids

Media was collected 48 hours after culture and stored at -80°C until estradiol (E2) and progesterone (P) determination using chemiluminescence by Immunoassay System (Siemens Healthcare Diagnostics Inc., Deerfield, United States). The lower limits of detection for E2 and P were 73.4 pmol/L and 0.6 nmol/L, respectively.

### Real-time RT-PCR analysis

To study fractalkine regulation of steroidogenic enzymes, Sybrgreen-based quantitative RT-PCR was carried out to measure transcript levels of steroidogenic acute regulatory protein (StAR), 3-β hydroxysteroid dehydrogenase (3βHSD) and cytochrome P45011A (CYP11A). Isolated granulosa cells were treated with 20 ng/ml hCG in the presence or absence of 100 ng/ml fractalkine for 48 h. Cells were washed and collected for subsequent extraction of total RNA. Total RNA was extracted using the Trizol reagent (Invitrogen, Carlsbad, United States) according to the manufacturer's protocol, and DNA was removed using the Turbo DNA-free kit (Ambion, Austin, United States). Based on absorbance at 260 nm, RNA samples were adjusted to 1 μg/μl before reverse transcription was performed using Superscript reverse transcriptase (Invitrogen, Carlsbad, United States). Real-time PCR was performed in 20 μl reaction volumes containing 10 μl 2 × Brilliant SYBR Green quantitative PCR master mix (Applied Biosystems Inc, Foster City, USA), 2 μl template cDNA, 0.5 μM primers, and 300 nM reference dye. β-actin was used for normalisation; however, the reserve transcription reaction was omitted for the negative controls. Plasmids of StAR, 3 -βHSD, CYP11A and β-actin were amplified to generate standard curves after serial dilutions. Realtime RT-PCR results were analysed using the ABI Prism 7000 SDS software (Applied Biosystems Inc, Foster City, USA). The following primers were used:

StAR, sense, 5'-GAGCAGAAGGGTGTCATCAGG-3', antisense, 5'- GCAGGTGGTTGGCAAAATC-3'; 3 -βHSD, sense, 5'-CCAGCATCTTCTGTTTCCTGG-3', antisense, 5'-AGCTTGGTCTTGTTCTGGAGTTTAG-3'; CYP11A, sense, 5_ TGGAGTCGGTTTATGTCATCG -3_, antisense, 5'-GGCCACCCGGTCTTTCTT-3'.

### Statistical analysis

All data are presented as the mean ± SD of at least three independent experiments. The results were analysed by one-way ANOVA, and *P *< 0.05 was considered to be statistically significant.

## Results

### Expression of fractalkine and its receptor CX3CR1 in the human ovary

**Figure 1:**
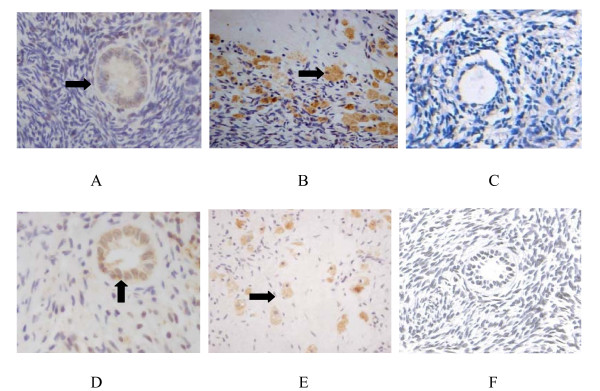
**Expression of fractalkine and CX3CR1 in the human ovary by immunohistochemistry**. A Fractalkine was localised in granulosa cells in the follicular phase (*arrowheads*). B Fractalkine was localised in luteinised granulosa cells (*arrowheads*). C Negative control (the primary antibodies were replaced by PBS). D CX3CR1 was localised in granulosa cells in the follicular phase (*arrowheads*). E CX3CR1 was localised in luteinised granulosa cells (*arrowheads*). F Negative control (the primary antibodies were replaced by PBS).

Immunohistochemical analysis from pathological specimens from 5 individuals demonstrated that fractalkine was expressed in granulosa cells in the follicular phase in human ovaries. Stronger signals were detected in luteal tissues and luteinised granulosa cells. For CX3CR1 staining, comparable levels of signal were found in granulosa cells in the follicular and luteal phases. Unlike fractalkine, CX3CR1 expression did not vary in different phases of the cycle. No signal was detected in control sections when primary antibodies were replaced by PBS. (Figure 1)

### Location of fractalkine and CX3CR1 in human luteinised granulosa cells

**Figure 2:**
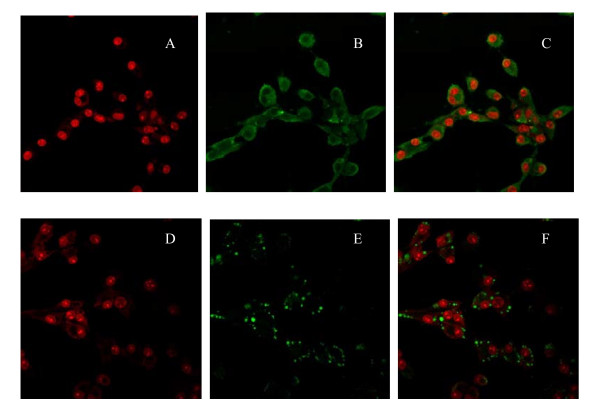
**Location of fractalkine and CX3CR1 in human luteinised granulosa cells by immunofluorescence with polyclonal rabbit anti-mouse/human/rat fractalkine and polyclonal rabbit anti-mouse/human/rat CX3CR1**. A A red fluorescent signal represents the nuclei of human luteinised granulosa cells. B A green fluorescent signal represents that fractalkine was located in the cytoplasm of human luteinised granulosa cells. C Composition graph of A and B. D A red fluorescent signal represents the nuclei of human luteinised granulosa cells. E A green fluorescent signal represents that CX3CR1 was located in the membrane of human luteinised granulosa cells. F Composition graph of D and E.

Observation using a confocal laser scanning microscope showed that fractalkine was distributed in the cytoplasm of human luteinised granulosa cells, and CX3CR1 was expressed in the membranes of human luteinised granulosa cells (Figure 2).

### Progesterone and estradiol production in cultured human luteinised granulosa cells treated with hCG and fractalkine

**Figure 3:**
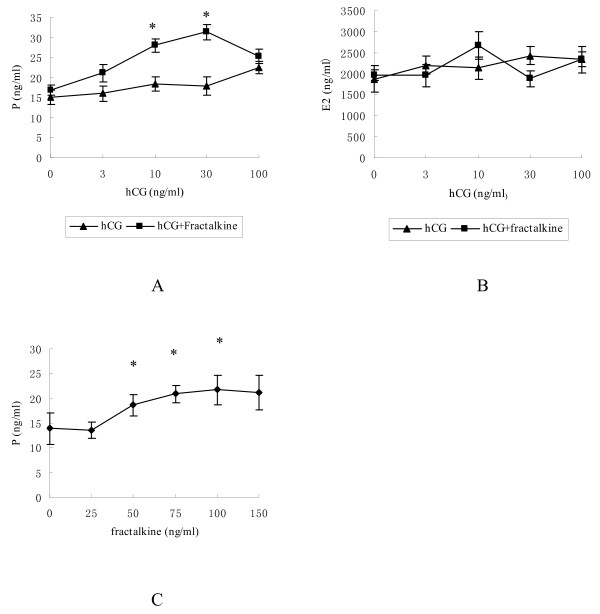
**The progesterone and estradiol contents in the medium of cultured human luteinised granulosa cells were determined using chemiluminescence**. A Progesterone content with graded doses of hCG (0, 3, 10, 30, 100 ng/ml) and with or without fractalkine (100 ng/ml); B Estradiol content with graded doses of hCG (0, 3, 10, 30, 100 ng/ml) and with or without fractalkine (100 ng/ml); C Progesterone content with graded doses of fractalkine (0, 25, 50, 75, 100, 150 ng/ml) and with hCG (20 ng/ml). *, *P *< 0.05 compared with corresponding cells treated with hCG alone.

To study the effects of fractalkine on steroidogenesis, we isolated and cultured human luteinised granulosa cells with graded doses of hCG in the presence or absence of recombinant fractalkine (100 ng/ml). After culturing for 48 h, media progesterone and estradiol levels were detected by chemiluminescence. As shown in Figure 3A, progesterone secretion was stimulated dose dependently by hCG (no statistical differences). When cells were cotreated with fractalkine and hCG, progesterone production was further increased. Although fractalkine increased hCG-stimulated progesterone production, no changes in media estradiol levels were found in the same cultures (Figure 3B).

The promoting effect of fractalkine on progesterone production was dose dependent. As shown in Figure 3C, cells were treated with 20 ng/ml hCG and increasing concentrations of fractalkine for 48 h. Progesterone biosynthesis was enhanced with graded doses of fractalkine.

### Regulation of steroidogenic enzymes induced by hCG and fractalkine in human luteinised granulosa cells

**Figure 4:**
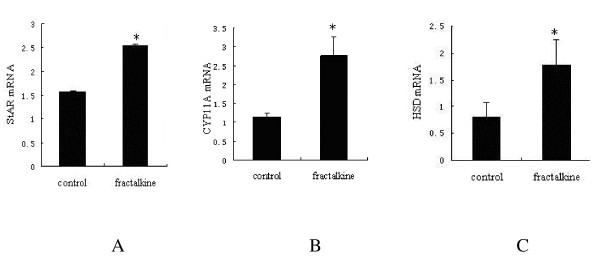
**The effect of fractalkine on the hCG stimulation of transcript levels for different steroidogenic enzymes in cultured granulosa cells**. Isolated granulosa cells were cultured with 20 ng/ml hCG and/or 100 ng/ml fractalkine for 48 h. Transcript levels of StAR (A), CYP11A (B) and 3β-HSD (C) were quantitated using real-time RT-PCR. Data are represented as the mean ± SE of three independent experiments. A Transcript levels of StAR; B Transcript levels of CYP11A; C Transcript levels of 3β-HSD. *, *P *< 0.05 compared to cells treated with hCG alone.

To investigate the mechanisms underlying fractalkine augmentation of hCG-stimulated progesterone production, we performed real-time RT-PCR of key steroidogenic enzymes StAR, CYP11A, and 3β-HSD. Based on the dose dependency of progesterone stimulation induced by fractalkine, 100 ng/ml of fractalkine were chosen. Human luteinised granulosa cells were treated with 20 ng/ml hCG and with or without 100 ng/ml fractalkine for 48 h. Real-time RT-PCR analyses (Figure 4) indicated that fractalkine cotreatment significantly increased the hCG stimulation of transcript levels for StAR, CYP11A, and 3β-HSD (*P *< 0.05).

## Discussion

Our results demonstrate for the first time that fractalkine and CX3CR1 are expressed in human ovarian tissues and human luteinising granulosa cells. Higher expression of fractalkine was found in luteinising granulosa cells than in granulosa cells in the follicular phase. Fractalkine can augment biosynthesis of progesterone in a dose-dependent manner but without affecting estradiol production by enhancing key steroidogenic enzymes in transcript levels. Increased expression of fractalkine/CX3CR1 can enhance progesterone production in luteinised human granulosa cells, which is consistent with the results of previous studies on rats [4]. To our knowledge, this is the first detailed report of fractalkine and CX3CR1 expression and the first description of their functions in human ovary and granulosa cells.

As previously described, CX3CR1 is a seven transmembrane-spanning G protein coupled receptor expressed on monocytes, NK cells, and some lymphocyte subpopulations [7]. CX3CR1 ligand (CX3CL1), also called fractalkine, is the sole member of the CX3C-chemokine subfamily. The soluble CX3CL1 exerts potent chemotactic activity [8]. In an *in vitro *model, fractalkine has been shown to have multiple activities, including signal transduction through the G protein-coupled receptor CX3CR1 and adhesion of monocytes, NK cells, and T cells in static binding assays [9,10].

Previous studies of fractalkine as a kind of chemokine were focused on its functions in adhesion and chemotaxis [11,12], while recent research paid more attention to the expression and function of chemokines in ovaries. Fractalkine was found to express in PMSG-primed rat ovary 6 h post-hCG [13]. Zhao et al. reported that fractalkine and CX3CR1 were found to express in the different ovarian compartments of rats, and treatment with fractalkine increased hCG stimulation of progesterone [4]. These data inspired us to study the function of fractalkine in the human ovary.

Our study showed that CX3CR1, the only fractalkine receptor, was expressed in human granulosa cells, and immunohistochemical analysis showed higher expression of fractalkine in luteinised granulosa cells than granulosa cells in the follicular phase; however, its receptor expression was not differential. This indicated that fractalkine had effects on human ovarian granulosa cells, especially in the luteinising physiological process. Due to the scarcity of human ovarian tissue, we chose granulosa cells derived from IVF-ET as materials in which to observe the location of fractalkine and CX3CR1. We found that fractalkine was expressed in the cytoplasm while CX3CR1 was expressed in the membrane of luteinised granulosa cells. This finding is consistent with classical ligand/receptor theory, which states that a ligand interacts with its receptor in the cell membrane and then plays its role elsewhere [14].

In our study, progesterone secretion was stimulated dose dependently by hCG in cultured luteinised granulosa cells. Previous studies have shown that IL-1B, prolactin and pituitary adenylate cyclase-activating polypeptide were found to be increased by the preovulatory LH/hCG surge and could promote progesterone biosynthesis and luteinisation [15-17]. In this study, progesterone production was further increased when cells were co-treated with fractalkine and hCG. Fractalkine significantly stimulated progesterone production in a dose-dependent manner. Higher expression of fractalkine was found in luteinised granulosa cells than in granulosa cells in the follicular phase. Above results indicated that fractalkine plays important roles in the ovary luteinising process as autocrine/paracrine factors. The finding that fractalkine could increase hCG-stimulated progesterone production may have clinical relevance in some reproductive endocrine diseases, such as corpus luteum function defect and polycystic ovary syndrome with insufficient progesterone, which results in menstrual disorder and abortion. Further study may lay the foundation for clinical treatment of these diseases.

To investigate the mechanisms underlying fractalkine augmentation of hCG-stimulated progesterone production, we measured three key steroidogenic enzymes levels. Real-time RT-PCR analysis showed that fractalkine cotreatment significantly augmented the hCG stimulation of transcript levels for StAR, CYP11A, and 3β-HSD. As well known, StAR plays an essential role in cholesterol transfer from the outer to the inner mitochondrial membrane, thus providing the substrate for steroid hormone biosynthesis. Cholesterol is then converted to pregnenolone by the CYP11A enzyme. 3β-HSD contributes to the conversion from pregnenolone to progesterone [18-21]. In our study, accompanied by increases in the levels of three key steroidogenic enzymes, progesterone synthesis is up-regulated by the synergistic effect of fractalkine and hCG. In a previous study, treatment with fractalkine was found to augment transcript levels for StAR, CYP11A, and 3β-HSD by increasing the phosphorylation of P38 MAPK in cultured granulosa cells of rats [4]. This needs to be verified in human granulose cells. We have to emphasize that we didn't try to directly demonstrate the existence of a functional fractalkine receptor on human granulosa cells in the current study. Therefore, further functional assays such as calcium mobilization, chemotaxis or MAPK kinase assays in human granulosa cells in response to fractalkine are needed to substantiate the function of the receptor.

It is interesting to note that no changes in estradiol production were found with the treatment of fractalkine compared with increased hCG-stimulated progesterone production in the same culture. Given the fact that estrogen is synthesised from progesterone by two pathways after ovulation, the relations between fractalkine and estrogen may be complicated by multiple factors and pathways. This is an issue deserving further investigation.

## Conclusions

Our study demonstrated for the first time that fractalkine and its receptor CX3CR1 are expressed in the human ovary. In addition, we showed that fractalkine could increase progesterone production through enhancing steroidogenic enzymes by fractalkine in luteinised human granulosa cells. These results build a solid foundation for further exploration of the roles of fractalkine in reproductive endocrine system and may have implications for the treatment of related reproductive diseases.

## List of abbreviations

A: androstenedione; CYP11A: cholesterol side chain cleavage; COH: controlled ovarian hyperstimulation; E2: estradiol; GC: granulosa cell; hCG: human Chorionic Gonadotrophin; 3 -βHSD: 3-β hydroxysteroid dehydrogenase; IVF-ET: in vitro Fertilisation-Embryo Transfer; LH: luteinising Hormone; P: progesterone; RT-PCR: reverse transcript-polymerase chain reaction; StAR: steroidogenic Acute Regulatory Protein

## Competing interests

The authors declare that they have no competing interests.

## Authors' contributions

SH carried out all of the experiments and drafted the manuscript. PZ participated in its design, carried out some work and helped to revise the manuscript. LY and YC participated in the immunohistochemistry and immunofluorescence. JY helped to collect human luteinised granulosa cells (GCs) from follicular aspirates. ED participated in the design of the study. JQ conceived of the study, participated in its design and coordinated and helped draft the manuscript. All authors read and approved the final manuscript.
